# The impact of blood flow restriction combined with variable resistance training at different loads on lower limb strength and explosiveness in collegiate basketball players

**DOI:** 10.3389/fphys.2025.1670071

**Published:** 2025-10-23

**Authors:** Wuwen Peng, Zhanming Zhang, Di Lu, Zhentao Li, Guoxing Li, Jian Sun, Duanying Li

**Affiliations:** ^1^ School of Athletic Training, Guangzhou Sport University, Guangzhou, Guangdong, China; ^2^ School of Physical Education, Guangzhou Sport University, Guangzhou, Guangdong, China; ^3^ Mumianwan School, Shenzhen, Guangdong, China; ^4^ Key Laboratory of Human-Computer Intelligent Interaction for Athletic Performance and Health Promotion, Guangzhou, Guangdong, China

**Keywords:** blood flow restriction training, chain variable resistance training, college male basketball player, lower limb sports performance, athletic performance

## Abstract

This study compared the effects of an 8-week program of low-load blood flow restriction training combined with chain-based variable resistance training (BFR-VRT) and high-load chain-based variable resistance training (VRT) on lower-limb performance in male college basketball players. A total of 27 players were randomly assigned to the BFR-VRT group (n = 9), the variable resistance training group (n = 9), or a control group (n = 9). The BFR-VRT protocol used pneumatic cuffs applied to the proximal thighs, with training pressure set at 180 mmHg, and combined a 10% 1RM barbell load with 10%–30% 1RM from chains, whereas the VRT protocol consisted of a 55% 1RM barbell load plus 10%–30% 1RM from chains. The control group did not receive any resistance training. Repeated-measures Repeated-measures ANOVA showed significant within-group improvements in SQ-1RM, LTC, RTC, countermovement jump, squat jump, DJ-RSI, standing long jump, and 30-m in both the BFR-VRT and VRT groups (all p < 0.05), whereas the CON group showed no significant change (p > 0.05). Between-group comparisons indicated no significant differences among the three groups on these outcomes (all p > 0.05). In conclusion, both BFR-VRT and VRT improved lower-limb performance; under a lower external load, BFR-VRT showed effects comparable to, or trending greater than, VRT on certain performance measures.

**Clinical Trial Registration:**

https://www.chictr.org.cn/showprojEN.html?proj=201896, identifier ChiCTR2400086132.

## 1 Introduction

Basketball is a team sport that requires athletes to have strong physical abilities (Puente et al., 2017). During the game, athletes need to demonstrate their athletic skills (such as confrontation, jumping, sprinting, and changing direction) to achieve victory (Ramirez-Campillo et al., 2022; Stojanović et al., 2018; Scanlan et al., 2015). A variety of resistance training methods are often used in basketball strength and conditioning (Archer et al., 2016). In addition to traditional strength training, other training methods such as variable resistance training (Lorenz, 2014) (VRT) and blood flow restriction training (Scott et al., 2015) (BFRT) can improve muscle strength and explosive power (Chaouachi et al., 2009; Hoffman, 2011).

The VRT involves attaching chains to both ends of the barbell. During the squat, part of the chains rest on the ground, while as the lifter stands up, the chains are gradually lifted off the ground, thereby increasing resistance throughout the range of motion. This variable loading allows athletes to complete the movement at a greater speed (Frost et al., 2010),inducing neuromuscular adaptation to improve muscle strength and movement performance. In high-load constant resistance training, the presence of the “StickingRegion” results in a deceleration phase during the final portion of the concentric squat movement (Elliott et al., 1989; Findley, 2004). Chains provide variable resistance that helps athletes overcome this deceleration, enabling them to exert greater force throughout the lift, thereby delivering a higher load and stronger training stimulus (Lander et al., 1985). Previous studies have shown that VRT may provide greater training stimulus than traditional free weight resistance training for increase strength and explosive power (B et al., 2011). Therefore, chain-based variable resistance training represents a superior option for trained individuals and athletes (Berning et al., 2008). Joy et al. (2016) confirmed this perspective, conducting a comparative study between chain-based VRT and constant resistance training. The results demonstrated that chain-based VRT enhances both maximal strength and vertical jump performance.

BFRT is an auxiliary training method that utilizes a specialized pressure device to partially restrict arterial inflow (typically 50%–80%) and venous return, thereby creating an ischemic and hypoxic environment. This approach allows low-load training to achieve adaptations comparable to those of high-load resistance training (Fujita et al., 2008). In recent years, as a new technology, BFR -combined with resistance training has been widely applied in sports such as rugby (Takarada et al., 2004), volleyball (Bagheri et al., 2018), track and field (Abe et al., 2005). Studies have confirmed that the high metabolic stress of BFRT, together with stimulation of the neuromuscular system,can promote the secretion of anabolic hormones, thereby facilitating muscle hypertrophy, strength development, and fatigue recovery (Flocco and Galeoto, 2021; Flocco et al., 2021). Castilla-López and Romero-Franco (2023) conducted a comparative study between low-load BFRT (L-BFRT) and high-load resistance training (HRT) involving 18 male athletes from the American Professional Football League. The results showed no significant difference in sprint performance improvement between the two groups. Therefore, L-BFRT is considered a meaningful alternative to HRT (Yasuda et al., 2008).

It is still controversial whether chain-based variable resistance training can improve explosive performance more effectively than constant resistance training. Some studies have shown that chain-based VRT alone may not be the most effective method for developing explosive powe (Archer et al., 2016). VRT and BFRT are widely used in various sports, but their mechanisms of enhancing performance differ. Therefore, it is worth exploring whether combining VRT with BFRT could produce superior outcomes.

In summary, this study designed an experiment study of BFRT combined with VRT of iron chains to explore the effects of low-load BFR-VRT and high-load VRT interventions on lower extremity motor performance.

## 2 Materials and methods

### 2.1 Study design

A randomized controlled trial was designed to investigate the effect of BFR-VRT on lower extremity performance of college male basketball players. After a familiarization session and baseline testing, participants were randomly assigned to the BFR-VRT, VRT, and CON groups, training twice per week for 8 weeks. In addition to the intervention, all players completed three basketball-specific technical practice sessions per week on Monday, Wednesday, and Friday, while intervention sessions were held on Tuesday and Saturday. Assessments were performed 1 week before and 1 week after the intervention.

The primary outcomes were back-squat one-repetition maximum (SQ-1RM) and countermovement jump (CMJ) height. The secondary outcomes (exploratory) included squat jump (SJ) height, drop-jump reactive strength index (DJ-RSI), standing long jump (SLJ) distance, 30-m sprint time, and left and right thigh circumference (LTC, RTC). All tests were conducted at least 48 h after the most recent training session. All tests and training sessions were conducted at the same facility under the direct supervision of the principal investigator. Prior to the formal intervention, participants completed a familiarization course to understand the overall study protocol and the test procedures. One additional testing session was held both before and after the intervention, which included lower extremity maximal strength, jump, and sprint assessments.

All training sessions were conducted under continuous, in-person supervision by study staff. Participants were explicitly instructed to report immediately any unusual sensations or adverse symptoms, including excessive pain, numbness/tingling, dizziness, or skin irritation at the cuff site. Any such symptoms were documented and evaluated, and training was paused or medical attention provided as appropriate.

### 2.2 Participants

The required sample size was calculated using G*Power 3.1 software, yielding a minimum of 27 participants based on an effect size (ES) of 0.6, an alpha level (α) of 0.05, and a statistical power of 0.95 (Lakens, 2022; Lin et al., 2022; Geng et al., 2024). To account for an anticipated 10% dropout rate, this study recruited 30 male college basketball players from Guangzhou Institute of Sport. The inclusion criteria of the subjects were: 1) healthy and free of cardiovascular disease; 2) The athletes have participated in basketball competitions above the provincial level and won the ranking; 3) No prior experience with BFRT; 4) More than 2 years of strength training experience; 5) No lower limb injury within the past 6 months. Participants missing two or more training sessions or sustaining an injury during the intervention were excluded from the analysis.

**FIGURE 1:**
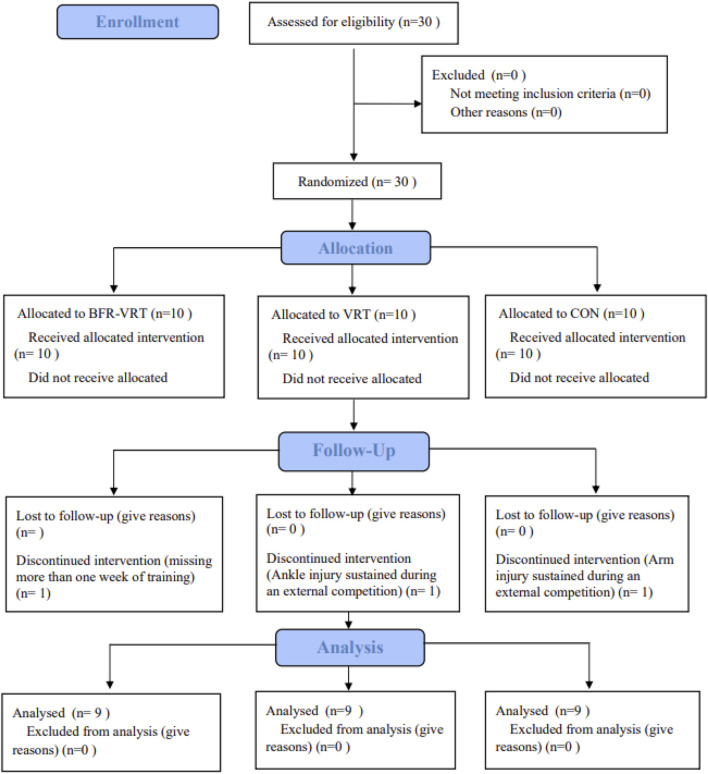
Random allocation process diagram.

After the familiarization session and baseling testing, participants were randomly assigned to the BFR-VRT, VRT, or CON groups (Figure 1). Allocation concealment was implemented using sequentially numbered, opaque, sealed envelopes prepared by an independent researcher before study initiation and opened after baseline assessments by a staff member not involved in outcome evaluation. Given the visible nature of the interventions, participants and training staff were not blinded; however, outcome assessors and the data analyst were blinded to group assignment. During the intervention period, three participants dropped out: one due to missing more than 1 week of training, and two due to ankle or arm injuries sustained during external competitions unrelated to the study. Consequently, 27 participants completed the trial and were included in the final analysis (BFR-VRT: n = 9; VRT: n = 9; CON: n = 9). Participant characteristics are presented in Table 1, and no significant differences were observed at baseline between the groups (p > 0.05). All participants were informed of the purpose and procedures of the study prior to the intervention and voluntarily agreed to participate. All procedures involving human subjects were conducted in accordance with the Declaration of Helsinki. Written informed consent was obtained from each participant after they were fully informed of the experimental procedures and potential risks. The study was approved by the Human Research Ethics Committee (Approval No. 2023LCLL-39) and prospectively registered with the Chinese Clinical Trial Registry (ChiCTR2400086132) on 25 June 2024, 2 days before enrollment of the first participant (27 June 2024).

**TABLE 1:** Basic information of athletes.

	BFR-VRT (n = 9)	VRT (n = 9)	CON (n = 9)	F	P
Age (y)	19.30 ± 0.80	19.89 ± 1.05	19.78 ± 1.09	0.764	0.477
Height (cm)	182.22 ± 2.72	186.78 ± 4.14	182.89 ± 7.13	2.165	0.137
Weight (kg)	78.11 ± 9.82	80.67 ± 9.32	74.78 ± 12.58	0.688	0.512
RTC	53.08 ± 3.90	52.28 ± 2.26	52.83 ± 3.53	0.137	0.872
LTC	53.02 ± 3.89	52.26 ± 2.34	52.92 ± 3.38	0.142	0.869
Squat 1RM (kg)	128.89 ± 16.15	130.00 ± 12.24	128.89 ± 10.54	0.021	0.979

### 2.3 Intervention

#### 2.3.1 BFR-VRT protocol

After a standardized warm-up, pneumatic cuffs (KAATSU Master, Sato Sports Plaza Inc., Japan) were applied circumferentially to the proximal region of both thighs (upper one-third, just below the inguinal crease). The inflatable bladder width of the cuffs was 6.5 cm, measured at the midpoint of the cuff. To ensure stable placement during training, a light holding pressure of approximately 30–40 mmHg was maintained during inter-set rest periods, as recommended in previous BFR protocols (Zhang et al., 2025). Early KAATSU methodology emphasized a conservative initiation and gradual progression in experienced users, with lower-limb pressures of 160–200 mmHg frequently reported in healthy adults (Takano et al., 2005). In line with contemporary safety guidance, a pressure of 180 mmHg was selected to balance participant safety with sufficient training stimulus (Patterson et al., 2019). It should be noted that this study did not individualize cuff pressure to each participant’s limb occlusion pressure (LOP). Under a fixed pressure of 180 mmHg, inter-individual differences in thigh circumference may lead to inconsistent relative occlusion levels (%LOP),participants with larger circumferences tend to experience a lower %LOP (potential under-dosing), whereas those with smaller circumferences may experience a higher %LOP (potential over-dosing) (Evin et al., 2021). The external load was prescribed as a barbell set at 10% 1RM plus bilateral chains calibrated so that, at the top position, the suspended chain mass equaled 10%–30% 1RM, while at the bottom position (parallel-thigh depth) the chains were largely deloaded onto the floor. Four sets were performed (30, 15, 15, 15 repetitions) with 1-min rest intervals between sets (see Table 2) (McMaster et al., 2009).

**TABLE 2:** Eight week experimental intervention plan.

	BFR-VRT	VRT	CON
Groups	4	4	Not
Times	30, 15, 15, 15	10, 10, 8, 8	Not
Intensity	10% + (10–30)%	55% + (10–30)%	Not
Intermittent	60 s	2 min	Not
Action	BFR + Chain Squat	Chain Squat	Not

BFR-VRT, Barbell load 10%1RM + Chain load (10%–30%) 1RM; VRT, Barbell load 55% 1RM + Chain load (10%–30%) 1RM; CON, without any intervention.

#### 2.3.2 VRT protocol

The barbell load was set at 55% 1RM, with chains calibrated so that, at the top position, the suspended chain mass equaled 10%–30% 1RM. Thus, the total load at the top ranged from approximately 65%–85% 1RM, while at the bottom it ranged from approximately 55%–65% 1RM. Four sets were performed (10, 10, 8, 8 repetitions) with 2-min rests (see Table 2).

#### 2.3.3 Iron chain configuration

Chains were attached to both ends of the barbell so that part of each chain rested on the floor at the bottom position. Each chain was 2.5 m in length, consisting of 25 links with a mass of about 1 kg per link (25 kg per side). Chain attachment and length were adjusted individually to ensure: (i) at the top position, the suspended chain mass corresponded to the planned percentage of 1RM; and (ii) at the bottom position (thighs parallel to the ground), most chain links were deloaded onto the floor (McMaster et al., 2009).

### 2.4 Testing procedures

**FIGURE 2:**
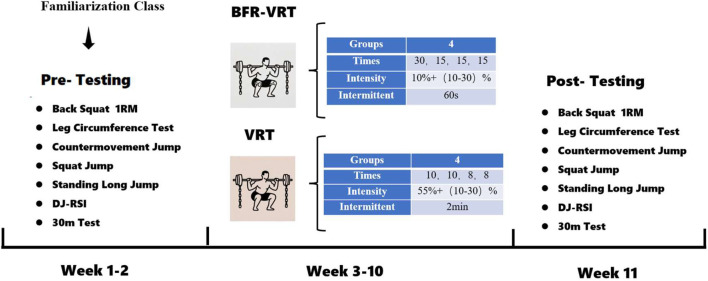
Experimental test flow chart.

Participants were tested before and after the formal start of the experiment, assessments were conducted over two nonconsecutive days separated by 48 h. On the first day, lower-extremity explosive performance was assessed, consisting of CMJ, SJ, DJ-RSI, SLJ, and 30m, and the second day was a TC and Squat-1RM, 48 h apart. Prior to all tests and training sessions, participants completed a standardized warm-up under supervision, which included 7 min of dynamic stretching and 3 min of core activation exercise. Post-intervention assessments were repeated 48 h after the final resistance-training session, as shown in Figure 2.

### 2.5 Outcome measures

#### 2.5.1 Squat-1RM

Maximum strength (1RM) is an important parameter to measure the basic strength level of athletes, and it is crucial for the athletic performance of athletes (Wisløff et al., 2004). After a standardized warm-up (1 set of 8–10 repetitions with an unloaded barbell), participants completed a progressive loading protocol: approximately 50% 1RM × 5 reps, 70% 1RM × 3–5 reps, 80% 1RM × 2 reps, 90% 1RM × 1 rep, then single attempts with incremental loads until failure. The last successful lift to parallel depth (thighs parallel to the floor), confirmed by two investigators, was recorded as 1RM. Rest intervals were standardized: 2 min between submaximal sets and 3 min before each 1RM attempt; failed attempts were permitted to be retried within 5 min (up to two additional attempts). Each participant completed four to six maximal attempts in total. For all repetitions, the eccentric phase was controlled and the concentric phase was performed at maximal intended velocity. Squat technique was standardized across warm-up, training, and testing sessions for all groups.

#### 2.5.2 Leg circumference test

All measurements were taken at the same time of day (morning) to minimize diurnal variation, and the same assessor conducted all left and right thigh circumference measurements. Participants wore form-fitting clothing; a non-elastic tape was held horizontally, snug but without compressing the skin, and values were recorded to the nearest 0.5 cm. For each thigh, three repeated measurements were obtained and averaged; if any reading differed by >5 mm, an additional measurement was taken and the three closest values were averaged for analysis.

#### 2.5.3 Vertical direction jump test (CMJ, SJ, DJ-RSI)

We included CMJ, SJ, and DJ-RSI as they are widely used in team-sport evaluations and commonly assess explosive strength under functional conditions, reflecting fundamental neuromuscular capacity (Ruggiero et al., 2022; Ruggiero and Gruber, 2024).

CMJ: During each attempt, perform a countermovement to a self-selected depth at a comfortable eccentric speed, then immediately perform a maximal vertical jump to reach the greatest possible height (no arm swing; hands on hips throughout).

SJ: Under the guidance of the researchers, the athlete lowers the body to a semi-squatting position and remains motionless for 2 s (no countermovement). After the 2-s hold, the athlete jumps maximally without any preparatory dip.

DJ-RSI: Participants stand on a 40-cm box positioned behind the contact mat and step off (do not jump down). Upon landing on the mat, they rebound as fast as possible into a vertical jump (minimize ground contact time). The contact mat recorded ground contact time and flight time, from which jump height was obtained; the system then reported RSI in the standard manner (jump height divided by contact time).

All jump tests were conducted using a mobile contact mat (SmartJump; Fusion Sport, Queensland, Australia) with a sampling frequency of 1,000 Hz. The system’s default sensitivity excludes flight times <100 m to prevent false triggering. Participants kept their hands on hips (no arm swing), aimed to fully extend the hips, knees, and ankles at take-off, and to land near the take-off point. Trials were deemed invalid if there was arm swing, an obvious preparatory dip in SJ, excessive horizontal displacement, or loss of balance. Each test comprised three maximal trials separated by 30–45 s; the best jump height (cm) was retained.

#### 2.5.4 Horizontal jump test (standing long jump)

Standing long jump was measured with a tape measure; chalk/powder was applied to the heels to mark the landing point. Each participant performed three maximal two-footed jumps with 2-min seated rests; the best distance was used for analysis. Participants started with feet shoulder-width and toes behind the take-off line, knees slightly flexed; at a verbal cue they used a natural arm swing and simultaneous two-leg push-off, fully extending the hips, knees, and ankles, and landed on both feet without stepping or falling. Distance was measured from the take-off line to the nearest heel mark; attempts were invalid if the line was crossed or balance was lost.

#### 2.5.5 30m sprint test

Sprint performance was assessed with dual-beam electronic timing gates (Brower Timing Systems, United States), the system consists of an active IR emitter (Photogate B) and receiver (Photogate A), which form a robust beam not reliant on a reflector and less affected by sunlight; gate alignment followed manufacturer procedures. The TCi Timer was set to 1/1000-s resolution (displaying milliseconds for short spans), with a specified timing accuracy of 0.001 s and radio switch accuracy of 0.0005 s. Each participant completed three maximal trials separated by 2-min seated rest, and the fastest time was retained. Athletes used a standing start with the lead foot behind the start line and remained motionless until the verbal cue (“Run!”); any movement before the cue or lifting a foot constituted a false start and the trial was repeated after rest. Times were recorded to 0.01 s (e.g., 3.21 s); timing started when the start beam was broken and stopped when the finish beam was broken.

### 2.6 Statistical analyses

Statistical analyses were performed using SPSS Statistics, version 25.0 (IBM Corp., Armonk, NY, United States). Data are presented as mean ± standard deviation (M ± SD). Normality was assessed with the Shapiro–Wilk test, and homogeneity of variances with Levene’s test. A two-way repeated-measures ANOVA [group (BFR-VRT, VRT, CON) × time (pre, post)] was conducted to examine group × time interactions and main effects (Cormack et al., 2008). Sphericity was assessed using Mauchly’s test; Greenhouse–Geisser corrections were applied when violated. When significant effects were detected, Bonferroni-adjusted *post hoc* pairwise comparisons were performed, and statistical significance was set at p < 0.05 (two-tailed). The test-retest reliability of the RTC,LTC,CMJ, SJ, DJ-RSI, SLJ, and 30 m was evaluated using coefficients of variation (CV) (Cormack et al., 2008) and intraclass correlation coefficients (ICC) (Koo and Li, 2016) with 95% confidence intervals (CIs) (Table 3). An ICC value below 0.5 indicates poor reliability, while values between 0.5 and 0.75 indicate moderate reliability, values from 0.75 to 0.9 indicate good reliability, and values above 0.90 indicate excellent reliability. An CV less than 10% is regarded as reliable. ccPartial squared eta ( $\eta_{p}^{2}$ ) is a measure of the effect size of differences between groups of intervention effects that were calculated and considered small (0.01 ≤ $\eta_{p}^{2}$ ≤ 0.06), moderate (0.06 ≤ $\eta_{p}^{2}$ < 0.14), or large ( $\eta_{p}^{2}$ ≥ 0.14) (Cohen, 2013).

**TABLE 3:** Reliability indices (ICC with 95% CI) and CV for each measure.

Subjects	ICC	95% CI	CV
RTC	0.985	(0.967, 0.993)	6.06%
LTC	0.927	(0.844, 0.966)	5.99%
CMJ	0.924	(0.839, 0.965)	7.62%
SJ	0.951	(0.895, 0.978)	12.02%
DJ-RSI	0.969	(0.932, 0.986)	13.96%
SLJ	0.897	(0.784, 0.952)	6.70%
30M	0.843	(0.682, 0.926)	6.61%

## 3 Results

**FIGURE 3:**
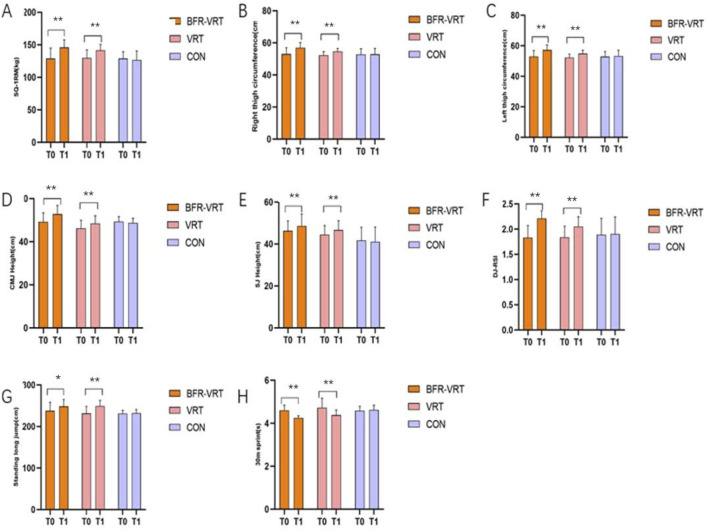
Changes in exercise performance during 8-week training. **(A)** SQ-1RM (kg); **(B)** right thigh circumference (cm); **(C)** left thigh circumference (cm); **(D)** countermovement jump (CMJ) height (cm); **(E)** squat jump (SJ) height (cm); **(F)** drop-jump reactive strength index (DJ-RSI); **(G)** standing long jump (m); **(H)** 30-m sprint time (s). *indicates the comparison between before and after the test, **indicates a very significant difference, P < 0.01.

Thirty athletes were recruited; due to three withdrawals during the intervention (missed sessions or injuries sustained in competitions outside the study), 27 participants were included in the final analyses. All completers attended 16/16 prescribed sessions (100% session-attendance adherence), and no intervention-related adverse events were observed during supervised training sessions (Table 4). For the pre-specified primary outcomes (SQ-1RM, CMJ) and secondary outcomes (SJ, DJ-RSI, SLJ, 30-m, LTC, RTC), each outcome was analyzed using a two-way repeated-measures ANOVA (Group × Time). To control the family-wise error rate (FWER = 0.05), the Holm–Bonferroni procedure was applied within each outcome family (primary family: two tests; secondary family: six tests). Subsequently, pairwise between-group comparisons (BFR-VRT vs. VRT, BFR-VRT vs. CON, VRT vs. CON) were conducted only for outcomes that remained significant after Stage-1 adjustment; within each outcome, the three comparisons were Bonferroni-adjusted. The Results and tables uniformly report adjusted p-values, partial η^2^ from the ANOVA, and Hedges’ g (95% CI) for the pairwise contrasts. For extremely small adjusted p-values that fall below the software’s display precision, values are presented as “<0.01,” with corresponding effect sizes provided to ensure interpretability. Detailed results are presented in Table 5 and Figure 3.

**TABLE 4:** Adverse events by training session.

Adverse events category	Definition	BFR-VRT (9)	VRT (9)	CON (9)	Total (27)
Dizziness/presyncope	Self-reported, observed unsteadiness	0/9	0/9	0/9	0/27
Numbness/tingling	Persistent during/after set	0/9	0/9	0/9	0/27
Petechiae/ecchymosis	Visible petechiae or bruising	0/9	0/9	0/9	0/27
Skin injury	Abrasion/blister/pressure mark requiring care	0/9	0/9	0/9	0/27
Device malfunction	Cuff/pump fault requiring termination	0/9	0/9	0/9	0/27

**TABLE 5:** Changes in exercise performance during 8-week training.

Test	Group	T0	T1	Change %	ES (Lower, Upper)	Main and interaction effects	Pairwise multiple-comparisons
SQ-1RM	BFR-VRT	128.89 ± 16.15	146.11 ± 11.39^a^	13.93%	−1.39 (−2.26,−0.51)	Time: p < 0.01, $\eta_{p}^{2}$ = 0.65	BFR-VRT vs. VRT: p = 1.00, ES = 0.13 (−1.03, 1.30)
	VRT	130.00 ± 12.24	141.67 ± 9.01^a^	9.32%	−0.94 (−1.68, −0.20)	Group: p = 0.20, $\eta_{p}^{2}$ = 0.13	VRT vs. CON: p = 0.50,ES = 0.65 (−0.54, 1.84)
	CON	128.89 ± 10.54	126.67 ± 13.91	−1.77%	0.18 (−0.43, 0.78)	Interaction: p < 0.01, $\eta_{p}^{2}$ = 0.61	BFR-VRT vs. CON: p = 0.29, ES = 0.78 (−0.42, 1.98)
RTC	BFR-VRT	53.08 ± 3.90	56.94 ± 3.30^a^	7.38%	−1.21 (−1.86, −0.55)	Time: p < 0.01, $\eta_{p}^{2}$ = 0.84	BFR-VRT vs. VRT: p = 0.93, ES = 0.48 (−0.73, 1.70)
	VRT	52.28 ± 2.26	54.65 ± 2.00^a^	4.58%	−0.74 (−1.22, −0.26)	Group: p = 0.35, $\eta_{p}^{2}$ = 0.08	VRT vs. CON: p = 1.00, ES = 0.19 (−1.02, 1.39)
	CON	52.83 ± 3.53	52.92 ± 3.69	0.15%	−0.03 (−0.36, 0.30)	Interaction: p < 0.01, $\eta_{p}^{2}$ = 0.74	BFR-VRT vs. CON: p = 0.49, ES = 0.67 (−0.56, 1.89)
LTC	BFR-VRT	53.02 ± 3.89	57.23 ± 3.33^a^	8.06%	−1.31 (−2.01, −0.61)	Time: p < 0.01, $\eta_{p}^{2}$ = 0.86	BFR-VRT vs. VRT: p = 0.92, ES = 0.49 (−0.73, 1.70)
	VRT	52.26 ± 2.34	54.86 ± 2.23^a^	5.02%	−0.81 (−1.32, −0.30)	Group: p = 0.39, $\eta_{p}^{2}$ = 0.08	VRT vs. CON: p = 1.00, ES = 0.13 (−1.07, 1.33)
	CON	52.92 ± 3.38	53.37 ± 3.70	0.84%	−0.14 (−0.49, 0.21)	Interaction: p < 0.01, $\eta_{p}^{2}$ = 0.72	BFR-VRT vs. CON: p = 0.60, ES = 0.62 (−0.60, 1.84)
CMJ	BFR-VRT	49.25 ± 4.17	52.83 ± 4.04^a^	7.34%	−1.04 (−1.73, −0.36)	Time: p < 0.01, $\eta_{p}^{2}$ = 0.58	BFR-VRT vs. VRT: p = 0.09, ES = 1.07 (−0.18, 2.32)
	VRT	46.24 ± 3.73	48.50 ± 3.56^a^	5.00%	−0.66 (−1.23, −0.08)	Group: p = 0.09, $\eta_{p}^{2}$ = 0.19	VRT vs. CON: p = 0.88, ES = −0.49 (−1.69, 0.70)
	CON	49.37 ± 2.35	48.74 ± 2.18	−1.24%	0.19 (−0.31, 0.68)	Interaction: p < 0.01, $\eta_{p}^{2}$ = 0.59	BFR-VRT vs. CON: p = 0.66, ES = 0.58 (−0.62, 1.78)
SJ	BFR-VRT	46.33 ± 4.68	48.63 ± 5.70^a^	4.88%	−0.42 (−0.88, 0.05)	Time: p < 0.01, $\eta_{p}^{2}$ = 0.29	BFR-VRT vs. VRT: p = 1.00, ES = 0.35 (−0.85, 1.54)
	VRT	44.49 ± 4.30	46.65 ± 4.45^a^	4.96%	−0.39 (−0.85, 0.07)	Group: p = 0.07, $\eta_{p}^{2}$ = 0.20	VRT vs. CON: p = 0.35, ES = 0.75 (−0.47, 1.98)
	CON	41.74 ± 6.25	41.14 ± 7.00	−1.62%	0.11 (−0.32, 0.53)	Interaction: p = 0.02, $\eta_{p}^{2}$ = 0.30	BFR-VRT vs. CON: p = 0.08, ES = 1.10 (−0.16, 2.36)
DJ-RSI	BFR-VRT	1.83 ± 0.24	2.21 ± 0.15^a^	21.79%	−1.50 (−2.28, −0.71)	Time: p < 0.01, $\eta_{p}^{2}$ = 0.87	BFR-VRT vs. VRT: p = 1.00, ES = 0.30 (−0.90, 1.51)
	VRT	1.84 ± 0.22	2.05 ± 0.19^a^	12.14%	−0.85 (-1.37, −0.32)	Group: p = 0.57, $\eta_{p}^{2}$ = 0.05	VRT vs. CON: p = 1.00, ES = 0.19 (−1.01, 1.39)
	CON	1.89 ± 0.32	1.90 ± 0.33	0.76%	−0.06 (−0.41, 0.29)	Interaction: p < 0.01, $\eta_{p}^{2}$ = 0.79	BFR-VRT vs. CON: p = 0.90, ES = 0.49 (−0.72, 1.70)
SLJ	BFR-VRT	237.89 ± 20.46	248.33 ± 16.51^b^	4.63%	−0.71 (−1.45, 0.03)	Time: p < 0.01, $\eta_{p}^{2}$ = 0.57	BFR-VRT vs. VRT: p = 1.00, ES = 0.17 (−0.98, 1.33)
	VRT	231.67 ± 16.84	249.44 ± 13.64^a^	7.88%	−1.21 (−2.08, −0.34)	Group: p = 0.23, $\eta_{p}^{2}$ = 0.12	VRT vs. CON: p = 0.61, ES = 0.59 (−0.59, 1.76)
	CON	231.22 ± 7.85	232.67 ± 8.36	0.64%	−0.10 (−0.76, 0.57)	Interaction: p < 0.01, $\eta_{p}^{2}$ = 0.38	BFR-VRT vs. CON: p = 0.31, ES = 0.76 (−0.43, 1.95)
30m	BFR-VRT	4.60 ± 0.23	4.24 ± 0.10^a^	−7.63%	1.37 (0.004, 2.74)	Time: p < 0.01, $\eta_{p}^{2}$ = 0.37	BFR-VRT vs. VRT: p = 0.60, ES = −0.50 (−1.50, 0.49)
	VRT	4.72 ± 0.44	4.38 ± 0.23^a^	−6.61%	1.32 (−0.04, 2.68)	Group: p = 0.19, $\eta_{p}^{2}$ = 0.13	VRT vs. CON: p = 1.00, ES = −0.19 (−1.18, 0.79)
	CON	4.58 ± 0.19	4.62 ± 0.22	0.78%	−0.14 (−1.36, 1.09)	Interaction: p = 0.02, $\eta_{p}^{2}$ = 0.28	BFR-VRT vs. CON: p = 0.24, ES = −0.70 (−1.71, 0.32)

Abbreviations: SQ-1RM, squat-one repetition maximum; RTC, right thigh Circumference; LTC, Left thigh Circumference; CMJ, countermovement jump; SJ, squat jump; DJ-RSI, drop-jump reactive strength index; SLJ, standing long jump.

^a^ Indicates a very significant difference, P < 0.01.

^b^ Indicates the comparison between before and after the test.

### 3.1 Training loads

Training loads were anchored to baseline 1RM. In the BFR-VRT group, the average external load per repetition was 38.7 ± 4.8 kg (bottom: 25.8 ± 3.2 kg; top: 51.6 ± 6.5 kg). With 75 repetitions per session, the per-session volume-load was 2,900 ± 364 kg. In the VRT group, the average external load per repetition was 97.5 ± 9.2 kg (bottom: 84.5 ± 8.0 kg; top: 110.5 ± 10.4 kg); with 36 repetitions per session, the per-session volume-load was 3,510 ± 331 kg. Across 16 sessions, the cumulative volume-load was 46,400 ± 5,817 kg for BFR-VRT and 56,160 ± 5,291 kg for VRT. There was a significant difference between the two groups (p = 0.002, ES = 1.67 (0.56, 2.74)).

### 3.2 Squat-1RM

Repeated-measures ANOVA revealed a significant main effect of time on Squat-1RM across the three groups (p < 0.01, $\eta_{p}^{2}$ = 0.65), whereas the main effect of group was not significant (p = 0.202, $\eta_{p}^{2}$ = 0.13). A significant time × group interaction was observed (p < 0.01, $\eta_{p}^{2}$ = 0.61). Post-hoc comparisons showed significant within-group improvements in both the BFR-VRT group (p < 0.01, ES = 1.39) and the VRT group (p < 0.01, ES = 0.94), whereas no change was observed in the CON group (p = 1.00). Between-group comparisons showed no significant difference between BFR-VRT and CON (p = 0.29, ES = 0.78), no significant difference between VRT and CON (p = 0.50, ES = 0.65), and no significant difference between BFR-VRT and VRT (p = 1.00, ES = 0.13). In terms of effect size, both BFR-VRT and VRT exhibited moderate potential effects relative to the control group.

### 3.3 Leg circumference

For RTC, repeated-measures ANOVA revealed a significant main effect of time (p < 0.01, $\eta_{p}^{2}$ = 0.84), no significant main effect of group (p = 0.35, $\eta_{p}^{2}$ = 0.08), and a significant time × group interaction (p < 0.01, $\eta_{p}^{2}$ = 0.74). Post-hoc comparisons showed significant within-group improvements in both the BFR-VRT group (p < 0.01, ES = 1.21) and the VRT group (p < 0.01, ES = 0.74), whereas no change was observed in the CON group (p = 1.00). Between-group comparisons showed no significant difference between BFR-VRT and CON (p = 0.49, ES = 0.67), no significant difference between VRT and CON (p = 1.00, ES = 0.19), and no significant difference between BFR-VRT and VRT (p = 0.93, ES = 0.48). In terms of effect sizes, BFR-VRT exhibited a moderate potential effect relative to VRT and a small potential effect relative to the CON.

For LTC, repeated-measures ANOVA revealed a significant main effect of time (p < 0.01, $\eta_{p}^{2}$ = 0.86), no significant main effect of group (p = 0.39, $\eta_{p}^{2}$ = 0.08), and a significant time × group interaction (p < 0.01, $\eta_{p}^{2}$ = 0.72). Post-hoc comparisons showed significant within-group improvements in both the BFR-VRT group (p < 0.01, ES = 1.31) and the VRT group (p < 0.01, ES = 0.81), whereas no change was observed in the CON group (p = 1.00). Between-group comparisons showed no significant difference between BFR-VRT and CON (p = 0.60, ES = 0.62), no significant difference between VRT and CON (p = 1.00, ES = 0.13), and no significant difference between BFR-VRT and VRT (p = 0.92, ES = 0.49). In terms of effect sizes, BFR-VRT exhibited moderate and small potential effects relative to VRT and the CON.

### 3.4 Vertical direction jump

For CMJ, repeated-measures ANOVA revealed a significant main effect of time (p < 0.01, $\eta_{p}^{2}$ = 0.58), no significant main effect of group (p = 0.09, $\eta_{p}^{2}$ = 0.19), and a significant time × group interaction (p < 0.01, $\eta_{p}^{2}$ = 0.59). Post-hoc comparisons showed significant within-group improvements in both the BFR-VRT group (p < 0.01, ES = 1.04) and the VRT group (p < 0.01, ES = 0.66), whereas no change was observed in the CON group (p = 1.00). Between-group comparisons showed no significant difference between BFR-VRT and CON (p = 0.66, ES = 0.58), no significant difference between VRT and CON (p = 0.88, ES = −0.49), and no significant difference between BFR-VRT and VRT (p = 0.09, ES = 1.07). In terms of effect sizes, BFR-VRT demonstrated a large potential effect relative to VRT and a moderate potential effect relative to the CON.

For SJ, repeated-measures ANOVA revealed a significant main effect of time (p < 0.01, $\eta_{p}^{2}$ = 0.29), no significant main effect of group (p = 0.09, $\eta_{p}^{2}$ = 0.20), and a significant time × group interaction (p = 0.013, $\eta_{p}^{2}$ = 0.30). Post-hoc comparisons showed a trend toward within-group improvement in both the BFR-VRT group (p = 0.06, ES = 0.42) and the VRT group (p = 0.09, ES = 0.39), whereas no change was observed in the CON group (p = 1.00). Between-group comparisons showed no significant difference between BFR-VRT and CON (p = 0.08, ES = 1.10), no significant difference between VRT and CON (p = 0.35, ES = 0.75), and no significant difference between BFR-VRT and VRT (p = 1.00, ES = 0.35). In terms of effect sizes, BFR-VRT showed large and small potential effects relative to VRT and the control group, respectively, and VRT showed a moderate potential effect relative to CON.

For DJ-RSI, repeated-measures ANOVA revealed a significant main effect of time (p < 0.01, $\eta_{p}^{2}$ = 0.87), no significant main effect of group (p = 0.57, $\eta_{p}^{2}$ = 0.05), and a significant time × group interaction (p < 0.01, $\eta_{p}^{2}$ = 0.79). Post-hoc comparisons showed significant within-group improvements in both the BFR-VRT group (p < 0.01, ES = 1.50) and the VRT group (p < 0.01, ES = 0.85), whereas no change was observed in the CON group (p = 1.00). Between-group comparisons showed no significant difference between BFR-VRT and CON (p = 0.90, ES = 0.49), no significant difference between VRT and CON (p = 1.00, ES = 0.19), and no significant difference between BFR-VRT and VRT (p = 1.00, ES = 0.30). In terms of effect sizes, BFR-VRT exhibited small potential effects relative to both VRT and CON.

### 3.5 Horizontal jump

For SLJ, repeated-measures ANOVA revealed a significant main effect of time (p < 0.01, $\eta_{p}^{2}$ = 0.57), no significant main effect of group (p = 0.23, $\eta_{p}^{2}$ = 0.12), and a significant time × group interaction (p < 0.01, $\eta_{p}^{2}$ = 0.38). Post-hoc comparisons showed significant within-group improvements in both the BFR-VRT group (p < 0.01, ES = 1.50) and the VRT group (p < 0.01, ES = 0.85), whereas no change was observed in the CON group (p = 1.00). Between-group comparisons showed no significant difference between BFR-VRT and CON (p = 0.31, ES = 0.76), no significant difference between VRT and CON (p = 0.61, ES = 0.59), and no significant difference between BFR-VRT and VRT (p = 1.00, ES = 0.17). In terms of effect sizes, both BFR-VRT and VRT exhibited moderate potential effects relative to the control group.

### 3.6 30 m sprint

For the 30-m sprint, repeated-measures ANOVA revealed a significant main effect of time (p < 0.01, $\eta_{p}^{2}$ = 0.57), no significant main effect of group (p = 0.23, $\eta_{p}^{2}$ = 0.12), and a significant time × group interaction (p < 0.01, $\eta_{p}^{2}$ = 0.38). Post-hoc comparisons showed significant within-group improvements in both the BFR-VRT group (p < 0.01, ES = 1.50) and the VRT group (p < 0.01, ES = 0.85), whereas no change was observed in the CON group (p = 1.00). Between-group comparisons showed no significant difference between BFR-VRT and CON (p = 0.24, ES = 0.70), no significant difference between VRT and CON (p = 1.00, ES = 0.19), and no significant difference between BFR-VRT and VRT (p = 0.60, ES = 0.50). In terms of effect sizes, BFR-VRT exhibited moderate potential effects relative to both VRT and CON.

## 4 Discussion

### 4.1 Training loads

This study observed that the total training volume (external load) was higher in the VRT group than in the BFR-VRT group, indicating that the two groups were not matched for external dose. It should be noted, however, that external load is not equivalent to the body’s actual “internal stimulus.” The metric of sets × reps × load (kg lifted) primarily reflects mechanical work and does not directly capture physiological or tissue-level stress (Oliva Lozano et al., 2024). Moreover, differences in exercise tempo, inter-set rest, proximity to failure, and velocity loss can further weaken the correspondence between external and internal load. Therefore, external load alone is insufficient to determine whether the stimuli experienced by the two groups were equivalent (Impellizzeri et al., 2019).

In BFR training, the %LOP better indexes the magnitude of the internal stimulus (Patterson et al., 2019). For reasons of equipment and procedural feasibility, we used a fixed cuff pressure with continuous safety monitoring and also reported the distribution of thigh circumference to aid interpretation. It should be acknowledged that, under a fixed pressure, inter-individual differences in thigh girth, cuff width, and body position may introduce variability in %LOP (Evin et al., 2021),this constitutes a limitation of the present study.

Against this backdrop, BFR-VRT may still achieve strength and hypertrophy adaptations comparable to those of high-load training by increasing metabolic stress, lowering the recruitment threshold of fast-twitch fibers, and upregulating anabolic signaling (Chang et al., 2023). Complementarily, VRT distributes load more appropriately across the range of motion (relative deloading at the bottom and greater loading at the top), aligning with force–velocity and force–length relationships and generally favoring gains in maximal strength and power (Andersen et al., 2022). Given these mechanistic and programming differences, evaluation of “stimulus equivalence” should rely on functional outcome measures of the targeted capacities rather than on external load alone.

### 4.2 Maximum lower extremity strength and leg circumference

In this study, within-group analyses for SQ-1RM showed that after 8 weeks intervention both the BFR-VRT and VRT groups improved SQ-1RM and left and right leg circumference. Although the two groups trained at different external intensities, their overall training loads were comparable and were prescribed according to optimal BFRT loading (Scott et al., 2015), indicating that BFR-VRT achieved gains in maximal lower-limb strength comparable to those of high-load VRT despite the lower load. This approach may also lower the risk of load-related injury (Loenneke et al., 2012). A 6-week BFRT study in national-level powerlifters reported a 7.7% increase in the vastus lateralis, with no significant change in the non-BFR group. Importantly, the BFRT load in that study was only 30% 1RM, far below the 60%–85% 1RM used in high-load constant-resistance training. Increases in thigh circumference likely reflect hypertrophy—i.e., greater muscle-fiber cross-sectional area—following BFRT, which in turn contributes to greater maximal lower-limb strength (Bjørnsen et al., 2018).

The results of a meta-analysis on variable resistance training of iron chains (Soria-Gila et al., 2015) showed that trained individuals gained significantly more strength with VRT than with constant resistance training. The mechanisms by which VRT may increase maximal lower-limb strength include: (Puente et al., 2017): By design, chains increase load as leverage improves, maximizing force output across the full range of motion and thereby increasing muscular stimulus (Findley, 2004; Joy et al., 2016); (Ramirez-Campillo et al., 2022) In the process of variable resistance exercise, compared with constant resistance training, the linear increase or decrease of external load on the trainer can reduce the loss of speed (Swinton et al., 2011) and improving force at low movement speeds and explosive strength, thus improving the maximum strength (Walker et al., 2013; Stojanović et al., 2018). Compared with constant-resistance training, chain-based variable resistance training (VRT) elicited a higher peak movement velocity after 7 weeks (Ghigiarelli et al., 2009), and greater power output (Swinton et al., 2011), thereby generating greater force during the terminal portion of the concentric phase.

Recent reviews (Wortman et al., 2021) and meta-analysis (Flocco and Galeoto, 2021; Flocco et al., 2021) have shown that L-BFRT has a similar effect on SQ-1RM improvement as high-intensity resistance training, which proves that BFR is a suitable and feasible method to improve athletes’ maximum lower extremity strength. There are several reasons why low-load BFRT can achieve the effect of high-load training: (Puente et al., 2017): During the intervention, participants in the BFR-VRT group trained with pneumatic cuffs that partially limited arterial inflow and markedly restricted venous outflow to the exercising limb, promoting localized hypoxia and venous pooling; under these conditions, metabolites (e.g., inorganic phosphate, hydrogen ions, ADP) accumulate and intramuscular pH decreases—responses linked to metabolic stress and peripheral contractile impairment (Ruggiero et al., 2020). Concurrently, suppression of slow-twitch fiber contribution facilitates additional recruitment of fast-twitch fibers (Takarada et al., 2000),thereby augmenting strength development (Ramirez-Campillo et al., 2022). The resultant acidic milieu activates afferent receptors and signals the central nervous system, stimulating sympathetic activity and promoting the secretion of testosterone, growth hormone, and insulin-like growth factor (Reeves et al., 2006; Stojanović et al., 2018). BFRT has also been shown to increase neuronal nitric oxide synthase (nNOS) activity and nitric oxide (NO) concentrations, which are associated with upregulation of hepatocyte growth factor and suppression of myostatin (Pearson and Hussain, 2015). In turn, elevated hepatocyte growth factor activates quiescent muscle satellite cells, while reduced myostatin activity permits greater satellite-cell activation, leading to muscle-fiber hypertrophy and strength gains (Scanlan et al., 2015). BFRT can enhance the activity of protein-synthesis regulators (e.g., S6K1), activate the mammalian target of rapamycin complex 1 (mTORC1) signaling pathway, and stimulate muscle-protein synthesis (Loenneke et al., 2010), thereby contributing to strength improvements (Archer et al., 2016). Strength gains with BFRT are further attributed to increases in type II muscle-fiber cross-sectional area (Yasuda et al., 2005).

In summary, BFR combined with chain-based variable resistance training may enhance lower-limb strength because low-load BFR substantially increases metabolic stress; as lactate accumulates, the recruitment threshold of fast-twitch fibers decreases, enabling a greater contribution of fast-twitch fibers to force production. Concurrently, chain-based VRT augments neural drive—evidenced by increased motoneuron excitability and higher central action-potential firing frequencies (Folland and Williams, 2007),thereby recruiting more motor units and increasing muscle force. Together, these complementary adaptations likely underlie the superior improvements in maximal lower-limb strength observed with the combined intervention.

### 4.3 Lower extremity explosive power

In this study, both the BFR-VRT group and the VRT group significantly increased CMJ height, indicating improved concentric force production of the lower-limb extensors and a faster eccentric-to-concentric transition within the stretch–shortening cycle (SSC). The magnitude of improvement was greater in the BFR-VRT group, suggesting that low-intensity BFRT can sustain high power output and support high-velocity force production, thereby benefiting vertical jump performance. SJ performance improved significantly in both the BFR-VRT and VRT groups, and BFR-VRT exhibited a larger effect size, suggesting that cuffed low-load chain training may offer an advantage for developing pure concentric ability. For the DJ-RSI, both intervention groups exhibited large within-group gains, indicating that BFR-VRT has potential to enhance SSC mechanics; prior work has similarly shown that 8 weeks of BFRT improves drop-jump performance (Yang et al., 2022). Both the BFR-VRT and VRT groups improved SLJ performance, indicating better concentric force production of the lower-limb extensors and a more rapid eccentric-to-concentric transition; notably, the VRT group showed the larger improvement, suggesting a greater enhancement of horizontal explosive power. Both the BFR-VRT and VRT groups significantly improved 30-m sprint performance, with the BFR-VRT group showing the superior improvement, consistent with previous findings in male track-and-field athletes undergoing BFRT (Abe et al., 2005).

The reasons for the improvement of lower limb explosive power may include the following: (Puente et al., 2017): Variable resistance training increases motor-unit recruitment and optimizes the SSC, thereby enhancing the stretch reflex and accelerating force development (Aboodarda et al., 2014). The stretch reflex can elevate motor-unit activation and firing frequency, allow greater utilization of elastic energy stored in series elastic elements when fascicles remain quasi-isometric, and thus accelerate concentric contraction and augment force, together, these adaptations improve motor-unit recruitment capacity (Israetel et al., 2010; Ramirez-Campillo et al., 2022). Compared with constant resistance, variable resistance elicits higher extracellular signal-regulated kinase activation and greater acute elevations in circulating hormones after training; these responses may enhance fatigue resistance (Walker et al., 2013; Stojanović et al., 2018). During squatting, bottom-range deloading and top-range loading enable higher concentric velocities and a faster eccentric-to-concentric transition due to lower inertia. Eccentric-exercise–induced microdamage promotes myofibrillar remodeling during recovery (Brockett et al., 2001), improving maximal strength and explosiveness. Moreover, the early-concentric “sticking region” limits force transmission and high-threshold motor-unit recruitment; lower initial loads with BFR-VRT may mitigate this region, and strength-curve–matched loading permits higher bar velocities, facilitating recruitment of high-threshold motor units. Empirically, VRT increases velocity at the onset of the concentric phase, and this advantage carries through later in the lift (Baker and Newton, 2009; Scanlan et al., 2015). By the size principle, low-threshold slow-twitch fibers are recruited first, whereas high-threshold fast-twitch fibers require higher intensity (Henneman et al., 1965). BFRT, by creating hypoxic and metabolically stressful conditions, suppresses slow-twitch contribution and promotes additional recruitment of fast-twitch fibers (Takarada et al., 2000; Archer et al., 2016). Because explosive power is the product of force and velocity, increases in absolute strength translate into better explosive performance; BFRT promotes muscle hypertrophy and strength even at low external loads, which can be advantageous for lower-limb performance (Doma et al., 2020; Lorenz, 2014). Tendon stiffness of the lower limb is strongly associated with explosive power (American College of Sports Medicine, 2009). In their study, Centner et al. reported increased tendon stiffness after low-intensity BFRT combined with high-intensity resistance training, which may partially explain improvements in lower-limb performance (Centner et al., 2023).

According to the results of this experiment, progressive exposure to BFR-VRT produced increasingly pronounced gains in lower-limb explosive performance. As a nontraditional resistance modality, BFR-VRT adjusts external resistance to better match the human strength curve, thereby enhancing strength, movement speed, and explosive power, accordingly, it represents a practical, learnable option for athletic preparation.

## 5 Conclusion

1. After 8 weeks, low-load BFR-VRT and high-load VRT both effectively improved lower-limb performance in collegiate male basketball players.
2. Compared with VRT, BFR-VRT demonstrated greater overall improvements. Given its substantially lower external loads, this low-load combined approach may serve as a feasible alternative to high-load VRT.

## 6 Limitations and future research directions

### 6.1 Limitations

This study has several limitations. First, blood-flow restriction was applied using a fixed absolute pressure (180 mmHg) rather than individualized to limb occlusion pressure (LOP), which does not account for differences in limb circumference, cuff–limb ratio, and vascular responsiveness, and may have introduced variability in the actual occlusion stimulus. Second, external loads were anchored to baseline 1RM without individualized week-to-week progression or mid-intervention strength reassessment; as adaptation occurred, the relative training stimulus likely diminished. Third, regarding performance assessment, the straight-line 30-m test may not fully capture the sport-specific demands of basketball. Accordingly, we have clarified in the Limitations that future studies should include 5/10/20-m splits and basketball-specific repeated-sprint/change-of-direction tests (e.g., three-quarter-court or shuttle formats) to better reflect on-court speed characteristics. Taken together, future research should adopt LOP-based individualized pressures (e.g., 40%–80% LOP), implement progressive, individualized loading with periodic re-testing to adjust intensity, and incorporate the above sprint-testing refinements to better optimize and evaluate athletes’ adaptation. We again thank the reviewer for this insightful comment, which helped us delineate the appropriate scope of our measurement approach more clearly.

## Data Availability

The original contributions presented in the study are included in the article/supplementary material, further inquiries can be directed to the corresponding authors.
